# Rupture of the Fallopian Tube from Accumulation of the Catamenial Fluid

**Published:** 1841-08

**Authors:** 


*Rupture of the Fallopian Tube from accumulation of the Catamenial Fluid.*—"Dr. Munk was requested, early on Tuesday morning, October 24, 1837, to visit R. S., æt 18, who, on his arrival, (about an hour subsequently,) was evidently *in articulo mortis.* Her pulse was barely perceptible, but extremely rapid; the skin was covered with a cold and clammy perspiration; there were low muttering delirium; tracheal rattle; the facies Hippocratica; and a continual involuntary discharge of the alvine contents. She died in a few hours.

"Upon making some inquiries into the history of the case, I was informed that she had never menstruated; that about eighteen months previously, when the evolution of the external organs of generation and the mammæ rendered probable the speedy appearance of the catamenia, she suffered from headache, pains in the back and limbs, cold extremities, and a heavy dragging sensation in the pelvic region, with some bearing down pains. These symptoms, after a brief continuance, subsided, but returned in five or six weeks; again ceased, and then returned after a short interval. This state of things continued for three or four months, the symptoms upon each recurrence remaining longer, and becoming more urgent, whilst the intermission became shorter in duration, and less perfect; so that at last there was no intermission, but manifest exacerbations occurring every fourth or fifth week.

"In January, the lower part of the abdomen began to swell; a deep-seated tensive pain was felt in the pelvis; all her symptoms were aggravated, and there was in addition occasional vomiting. She still, however, kept about her usual employment, and took, by the direction of a neighboring practitioner, some medicines, which, from the description given, I presume consisted either wholly or in part of iron. Under this treatment she got rapidly worse. On Friday the 20th, when stooping, she felt, to use her own expression, something give way within her, and the swelling of the abdomen appeared to subside, as did likewise the dragging and tensive pain abovementioned. Towards evening she complained of diffused pain of the abdomen, which, by Saturday, had so far increased that she was unable to bear the slightest pressure. There was great heat of skin, headache, knees flexed upon the abdomen, some difficulty of breathing, contracted state of the features, nausea and vomiting, with difficulty of, and intense pain over the abdomen, on emptying the bladder. This condition continued until Monday, when she passed gradually into the state in which I found her on the Tuesday morning.

"*Autopsy twenty-six hours after death.*—On laying open the abdomen, I was surprised to find a large quantity of a dark red and thickish fluid (somewhat similar in appearance to blood, which had been for some considerable time effused) lying in the cavity of the peritoneum, and amounting, I should imagine, to twelve or fourteen ounces. The peritoneum, which was everywhere in contact with it, was stained of a reddish colour. On wiping away the fluid with which it was covered, there was an evident increase of vascularity, and in some parts the membrane was covered with a thin layer of coagulable lymph. I searched for the source of this sanguiniform fluid, but for a considerable time without effect. The uterus at length attracted attention; it was considerably larger than the ordinary size of a man's fist, but, nevertheless, flaccid. Upon opening it, I found four or five ounces of a similar fluid to that in the abdomen, contained within its cavity. The fallopian tubes were enormously distended; so much so that I could with ease pass the little finger in them. Close to the fimbriated extremity of the left tube, there was a fissure about two lines in length, with rugged edges, thus forming a free communication from the cavity of the uterus to that of the peritoneum; through this the fluid in question had evidently passed. The lining membrane of the uterus was of a slightly reddish colour, depending, as I imagine, on its contact with the contained fluid. On pursuing the examination, I found an obstruction to the passage of the finger or probe through the vagina. This was caused by the opposition of the walls of the canal, and their junction by firm cicatrization. This cicatrix was from half an inch to an inch in length, and contained a hard tough substance in many respects resembling cartilage. I could find nothing at all resembling the hymen."—*London Med. Gaz., March,* 1841.

